# The combination of serum insulin, osteopontin, and hepatocyte growth factor predicts time to castration-resistant progression in androgen dependent metastatic prostate cancer- an exploratory study

**DOI:** 10.1186/s12885-016-2723-1

**Published:** 2016-09-06

**Authors:** Farshid Dayyani, Amado J. Zurita, Graciela M. Nogueras-González, Rebecca Slack, Randall E. Millikan, John C. Araujo, Gary E. Gallick, Christopher J. Logothetis, Paul G. Corn

**Affiliations:** 1 Department of Genitourinary Medical Oncology, The University of Texas M.D. Anderson Cancer Center, Dan L. Duncan Building (CPB7.3476), 1515 Holcombe Blvd., Unit 1374, Houston, TX 77030 USA; 2 Department of Biostatistics, The University of Texas M.D. Anderson Cancer Center, Houston, TX USA

**Keywords:** Insulin, Osteopontin, Hepatocyte growth factor, Castration resistance, Metastatic prostate cancer, Prognostic marker

## Abstract

**Background:**

We hypothesized that pretreatment serum levels of insulin and other serum markers would predict Progression-free survival (PFS), defined as time to castration-resistant progression or death, in metastatic androgen-dependent prostate cancer (mADPC).

**Methods:**

Serum samples from treatment-naïve men participating in a randomized phase 3 trial of ADT +/- chemotherapy were retrospectively analyzed using multiplex assays for insulin and multiple other soluble factors. Cox proportional hazards regression models were used to identify associations between individual factor levels and PFS.

**Results:**

Sixty six patients were evaluable (median age = 72 years; median prostate surface antigen [PSA] = 31.5 ng/mL; Caucasian = 86 %; Gleason score ≥8 = 77 %). In the univariable analysis, higher insulin (HR = 0.81 [0.67, 0.98] *p* = 0.03) and C-peptide (HR = 0.62 [0.39, 1.00]; *p* = 0.05) levels were associated with a longer PFS, while higher Hepatocyte Growth Factor (HGF; HR = 1.63 [1.06, 2.51] *p* = 0.03) and Osteopontin (OPN; HR = 1.56 [1.13, 2.15]; *p* = 0.01) levels were associated with a shorter PFS. In multivariable analysis, insulin below 2.1 (ln scale; HR = 2.55 [1.24, 5.23]; *p* = 0.011) and HGF above 8.9 (ln scale; HR = 2.67 [1.08, 3.70]; *p* = 0.027) levels were associated with longer PFS, while adjusted by OPN, C-peptide, trial therapy and metastatic volume. Four distinct risk groups were identified by counting the number of risk factors (RF) including low insulin, high HGF, high OPN levels, and low C-peptide levels (0, 1, 2, and 3). Median PFS was 9.8, 2.0, 1.6, and 0.7 years for each, respectively (*p* < 0.001).

**Conclusion:**

Pretreatment serum insulin, HGF, OPN, and C-peptide levels can predict PFS in men with mADPC treated with ADT. Risk groups based on these factors are superior predictors of PFS than each marker alone.

## Background

Metastatic prostate cancer is initially highly treatable using androgen-deprivation therapy (ADT) that depletes gonadal sources of systemic testosterone [1]. Over time, however, these cancers eventually lose their responsiveness to ADT and become castration-resistant. While the median time to castration-resistant progression is 18 to 24 months, the duration of response is quite variable, with some men developing castration-resistant disease within 6 months of initiating ADT, while others remain responsive for 5 years or more. This variability reflects the underlying biologic heterogeneity of prostate cancer progression, a complex multi-genic process occurring principally in bone, the preferred site of prostate cancer metastases [2]. Multiple signaling pathways provide crosstalk between the epithelial and the stromal compartments within the bone microenvironment to enhance tumor growth, including the androgen receptor, tyrosine-kinases, and immune surveillance [1]. The transition from androgen-dependent to castration-resistant disease is a clinically notable event, as it signals the imminent lethal potential of advanced disease. Death from metastatic prostate cancer typically occurs within 24 to 48 months of developing castration-resistance.

At present, prognostic markers for patients newly diagnosed with metastatic androgen-dependent prostate cancer (mADPC) remain relatively limited. The most reliable and significant predictors of overall survival are the absolute prostate specific antigen (PSA) nadir after 6–8 months of ADT initiation and the time to PSA progression after the nadir [3, 4]. An obvious disadvantage of these variables is that they are not available when patients are initially diagnosed and treated. While several studies have associated pretreatment variables with the development of castration resistance and overall survival, none has yet become routinely incorporated into clinical practice or clinical research, in part due to the small retrospective nature of the data and/or lack of prospective validation of individual biomarkers. For example, pretreatment PSA kinetics (but not absolute PSA values) and number of circulating tumor cells have been shown to predict time to castration-resistant progression in patients with advanced disease [5, 6], and serum alkaline phosphatase levels, serum PSA to acid phosphatase ratio, and extent of disease (number of bone lesions and/or involvement of viscera) with overall survival [7–9]. Thus, as knowledge about the mechanisms of prostate cancer progression evolves, there is continued interest in developing novel candidate biomarkers that reflect the pathophysiology of the disease.

Of particular interest to our group has been the role of insulin metabolism and hyperinsulinemia in prostate cancer progression. In cells that express both the insulin receptor (IR) and insulin-like growth factor-1 receptor (IGF-1R), hybrid IGF-1R/IR receptors are formed, and these hybrid receptors are activated by insulin-like growth factor-1 (IGF-1) as well as likely insulin [10]. In a murine model, diet induced hyperinsulinemia has been shown to accelerate the growth of prostate cancer. Mice on a high carbohydrate-high fat diet had higher levels of serum IGF-1 and their tumors showed higher levels of activated Akt and higher insulin receptor levels than tumors from mice on a low carbohydrate-high fat diet [11]. Prostate cancer cell lines cultured in the presence of insulin induce steroidogenesis and increase their expression of PSA [12]. In addition, evidence from population-based screening cohorts has demonstrated that both insulin and IGF-1 levels correlate more closely than PSA levels with the risk of developing prostate cancer [11, 13]. Given evidence showing prostate cancer responsiveness to insulin in mouse models [11] and insulin receptor expression by human prostate cancer cells [14], it is possible that hyperinsulinemia facilitates progression to castration-resistant prostate cancer (CRPC) [15].

With this background, the goal of the current study was to explore whether pre-treatment levels of insulin and other biomarker candidates would predict Progression-free survival (PFS) defined as time to castration-resistant progression or death in men with mADPC initiated on ADT. The role of insulin metabolism in CRPC progression deserves further study because of the increasing prevalence of hyperinsulinemia in the population at risk to develop prostate cancer, and because of the availability of therapies (such as dual small molecule inhibitors of IGF-1R and IR) to target insulin and IGF-1 pathways in patients with advanced disease [16].

In the present study we retrospectively analyzed banked serum samples from a randomized phase 3 study evaluating whether chemotherapy given in addition to standard androgen deprivation would delay the appearance of castration-resistant disease. Study participants were randomized and time to emergence of CRPC, the primary endpoint, was not statistically different between groups, suggesting that the addition of chemotherapy to ADT does not confer a clinical benefit in this population [17]. The primary endpoint of the current study was to determine if the baseline insulin levels were associated with PFS. Secondary endpoints included the identification of other possible biomarkers for PFS.

## Methods

### Patients

Details regarding the eligibility criteria of included patients are published elsewhere [17]. In brief, 286 men with treatment-naïve metastatic prostate cancer were randomized to ADT alone or ADT plus ketoconazole and doxorubicin alternating with vinblastine and estramustine for three cycles. Castration-resistance was defined as disease progression with a serum testosterone level of <50 ng/dL. Both the primary endpoint of time to CRPC and the secondary endpoint of overall survival (OS) were not statistically different between the treatment arms [17]. For this reason, patients from both treatment arms were combined in the present analysis. All subjects provided informed written consent to participate in the phase III trial. A waiver of informed consent and authorization was previously approved to establish a historical tissue and data repository (protocol number LAB10-0335). Under this protocol, a modified honest broker system [18] was established to collect and provide coded health information to research investigators in a manner where it would not be reasonably possible for the investigators or others to identify the corresponding patients/specimens directly or indirectly. In this system, the honest broker holds the key to the patient’s identity and will in no circumstances release the codes. An additional waiver of informed consent and authorization was requested to allow laboratory testing to be performed as outlined in this protocol (protocol number LAB 10-0528), which was approved by the Institutional Review Board at MD Anderson. Serum analysis was approved by the Institutional Review Board at MD Anderson. The original phase III clinical study (internal MD Anderson trial ID: DM95-231) anteceded the introduction of the ClinicalTrials.gov website for trial registration in year 2000 and thus does not carry a trial registration number.

### Serum cytokine analysis

Non-fasting, pre-treatment serum samples from 66 patients were available for analysis. Circulating levels of soluble factors were measured using Searchlight immunoassays (Aushon BioSystems, Billerica, MA). Briefly, samples were incubated for 1 h on the array plates that were pre-spotted with capture antibodies specific for each protein biomarker. Plates were decanted and washed four times before adding a cocktail of biotinylated detection antibodies to each well. After incubating with detection antibodies for 30 min, plates were washed four times and incubated for 30 min with streptavidin-horseradish peroxidase conjugate. All incubations were done at room temperature with shaking at 200 rpm. Plates were again washed before adding a chemoluminescent substrate. The plates were immediately imaged using the Aushon Signature Plus CCD Imaging System©, and data was analyzed using Aushon PROarray Analyst Software©.

### Statistical analysis

PFS was defined as time from ADT treatment start to CRPC or death, whichever came first. CRPC was evidenced by any of the following: symptomatic or radiographic progression, increasing PSA, or receipt of any new systemic therapy with serum levels of testosterone <50 ng/ml [17]. Patients alive and free of progression at their last follow-up were censored on that date. Definition for disease volume was based on the original study, for our analysis patients were adjusted in high-volume metastasis (HVM, ≥3 bone lesions or visceral involvement) and low volume metastasis (LVM, <3 bone lesions and local/nodal involvement with or without prior definitive local therapy) [17]. Dates of PFS and death were independently confirmed by chart review by three of the authors (FD, JCA, PGC).

The Kaplan-Meier product limit method [19] was used to estimate the median PFS for each clinical/demographic factor and for each of the serum markers. Univariable Cox proportional hazards regression models [20] were used to identify any association with each of the variables and PFS. Serum markers were transformed using the natural log scale. If the transformed marker showed a skewed distribution to the right, the natural log of the serum marker was used for analysis. For each factor, medians, HRs, their 95 % confidence interval (CI), and hazard ratio *p*-values are presented in tables. Kaplan-Meier curves are presented for any significant factors associated with PFS. For serum marker measures, the univariable Cox proportional hazards regression *p*-value is based on the continuous measure, but for the Kaplan-Meier curves they are split at the median or using the values found using CART for presentation as described below. Multivariable Cox proportional hazards regression [20] adjusted by trial therapy and metastasis volume was used to model all the statistically significant variables in the univariable setting. Biomarkers were included as continuous values unless cumulative Martingale residual plots indicated transformation by cut-point indicator variables were more appropriate in the multivariable regression [21, 22]. CART analysis was used to identify biomarker cut points for the PFS analysis. The CART model included the biomarkers found statistically significant in the univariable analysis. Biomarkers with no identified cut point in the multivariable model were explored in a univariate CART analysis to determine whether any cut point could be identified since this is a first exploratory examination of these markers. Finally, we identified the number of risk factors for each patient by identifying whether a patient was above or below a cut point for each significant biomarker. If no cut point was found, then the biomarker was split by the median for the purposes of identifying whether a patient was on the risky side of the biomarker. Each patient was then given a score from 0 to the total number of significant biomarkers counting up how many of the markers the patient had on the poorer-performing direction. All comparisons used a two-sided significance level of 0.05 for this exploratory study to identify markers to be confirmed in a future study. A total of 55 biomarkers were tested, so only markers that are significant at <0.001 using Bonferroni correction are considered statistically significant without future confirmation. Statistical analysis was performed using STATA/SE version 13.1 statistical software (Stata Corp. LP, College Station, TX).

## Results

### Patients

Table 1 presents the patient characteristics of the population included in the analysis. The median age was 72 years and median PSA of was 31.5 ng/mL. Patients were primarily white (86 %), overweight/obese (59 %), without diabetes (94 %), with Zubrod performance status 1 or 2 (53 %), with high volume metastatic disease (HVM, 53 %), a Gleason score ≥8 (77 %), and were treated with ADT on trial (52 %).

**Table 1:** Patient characteristics

Characteristic	Number	Percent
All Patients	66	100
Age (years) Median (min, max)	72 (57, 93)	
PSA (ng/ml)—Median (min, max)	31.5 (1.0,64.0)	
Ethnicity		
Non-White	9	13.6
White	57	86.4
Body Mass Index		
Underweight (<18.5)	1	1.5
Normal (18.5–24.9)	13	19.7
Overweight (25.0–29.9)	25	37.9
Obese	14	21.2
Not Available	13	19.7
Diabetes		
No	62	93.9
Yes	4	6.1
Performance Status		
PS 0	31	47.0
PS1-2	35	53.0
Gleason score		
5–6	2	3.0
7	11	16.7
8–10	51	77.3
Missing	2	3.0
Metastatic Volume		
Low	31	47.0
High	35	53.0
Treatment Arm		
ADT	34	51.5
ADT + Chemo	31	47.0
Not Available	1	1.5

*ADT* Androgen Deprivation Therapy, *Chemo* Chemotherapy

### Progression-free survival

**Fig. 1:**
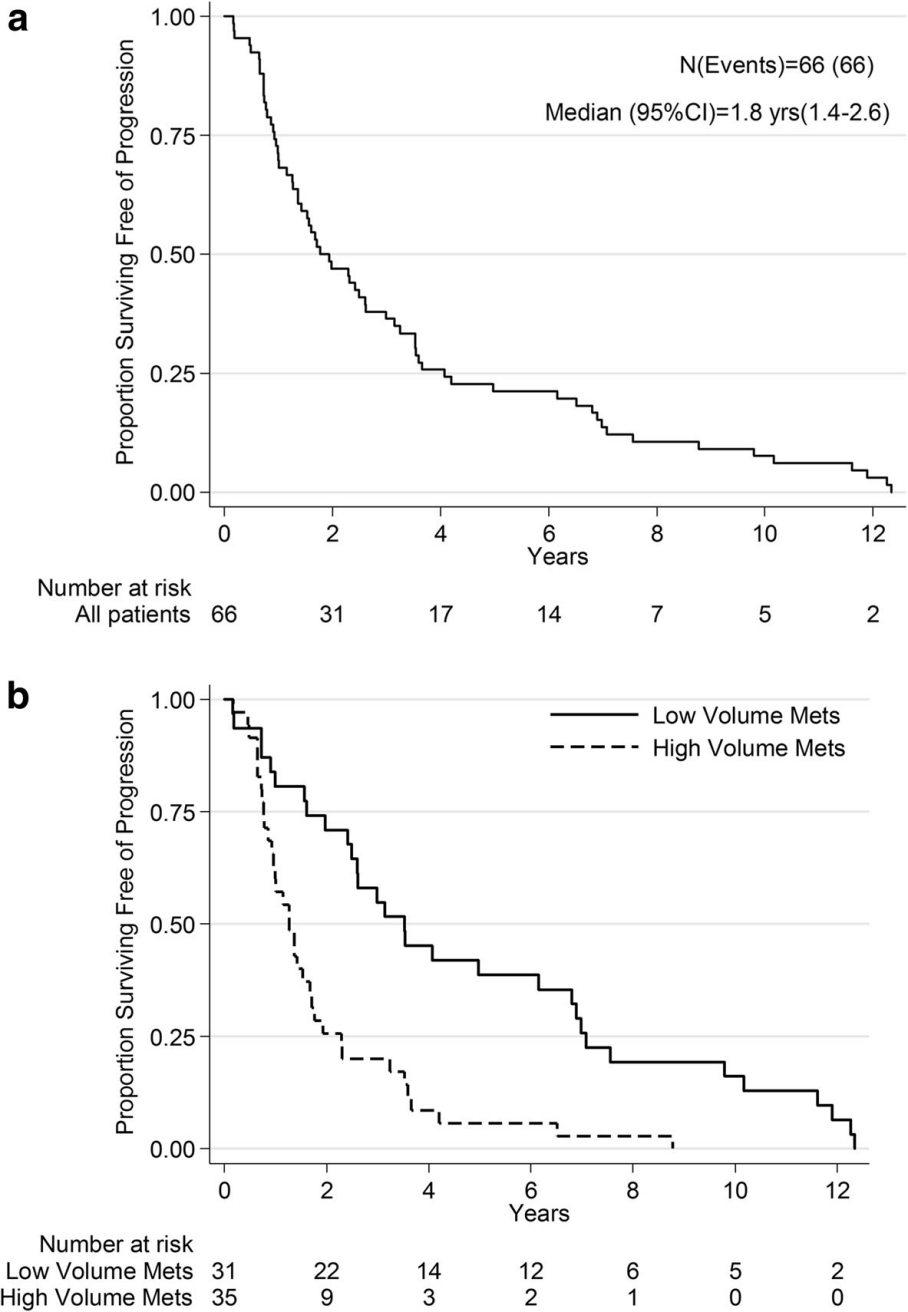
Progression-free survival (**a**) for all the patients and (**b**) by Metastasis Volume

**Fig. 2:**
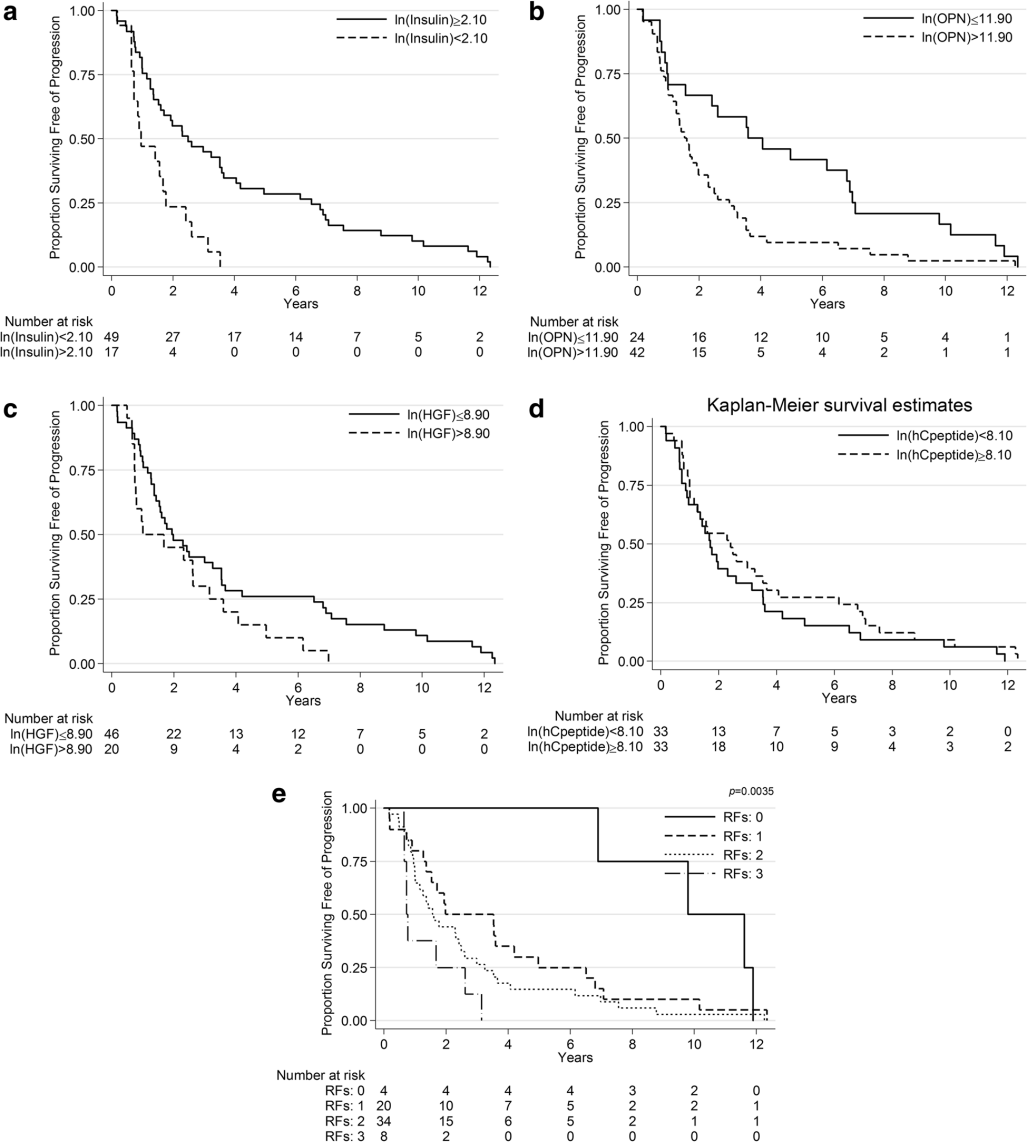
Progression-free Survival According to Risk Factors (RFs). (**a**) Low insulin (i.e. below CART cut-off), (**b**) high osteopontin (OPN), (**c**) high hepatocyte growth factor (HGF) (i.e. above CART cut off), and (**d**) low C-Peptide (i.e. below the median) each represent a risk factor (RF) for progression. (**e**) PFS curves were generated for patients falling in 3 separate risk groups: 0, 1, 2 and 3 RFs. No patients had 4 RF

Figure 1a presents the PFS for the group. The median PFS was 1.8 years (95 % CI: 1.4–2.6 years). Figure 1b presents the PFS curves by metastasis volume. Table 2 shows the univariable cox proportional hazard regression models for PFS. There were a total of 66/66 (100 %) progressions and/or deaths. Only metastatic volume, Insulin, C-peptide, Hepatocyte Growth Factor (HGF), and Osteopontin (OPN) were significantly associated with PFS. Patients with HVM were nearly three times as likely to develop castration-resistance compared to those with LVM (HR = 2.93 [1.70–5.05]; *p* = <0.001). Higher levels of Insulin (HR = 0.81, 95 % CI: 0.67–0.98; *p* = 0.03) and C-peptide (HR = 0.62, 95 % CI: 0.39–1.00; *p* = 0.05) were associated with longer PFS. Conversely, higher levels of HGF (HR = 1.63 [1.06–2.51]; *p* = 0.03) and OPN (HR = 1.56 [1.13–2.15]; *p* = 0.01) were associated with shorter PFS. Figure 2a-e presents the PFS curves for each of these factors.

**Table 2:** Progression-free survival results for patient characteristics and biomarkers

	N	No. Events	Median Years	HR	95 % CI for HR	HR *p*-value
Race						
Non-White	9	9	1.77			
White	57	57	1.93	1.20	(0.57, 2.54)	0.64
Gleason score						
5–7	13	13	3.59			
8–10	51	51	1.60	1.77	(0.94, 3.34)	0.08
Performance Status						
0	31	31	1.97			
1–2	35	35	1.70	1.20	(0.74, 1.96)	0.46
Metastatic Volume						
Low	31	31	3.53			
High	35	35	1.27	2.93	(1.70, 5.05)	<0.001
Treatment						
ADT	34	34	1.60			
ADT + Chemo	31	31	2.29	0.99	(0.60, 1.61)	0.95
Diabetes						
No	62	62	1.93			
Yes	4	4	0.92	2.28	(0.80, 6.45)	0.12
Age	66	66	1.77	0.99	(0.96, 1.02)	0.67
PSA (ln)	66	66	1.77	1.00	(0.99, 1.01)	0.65
Insulin (ln)	66	66	1.77	0.81	(0.67, 0.98)	0.03
RANK (ln)	66	66	1.77	0.82	(0.60, 1.12)	0.22
IGFBP3 (ln)	66	66	1.77	0.89	(0.52, 1.51)	0.66
IGFBP1 (ln)	66	66	1.77	1.05	(0.87, 1.27)	0.59
IGF I	66	66	1.77	0.85	(0.42, 1.74)	0.66
IGF II	66	66	1.77	0.80	(0.27, 2.42)	0.70
HGF (ln)	66	66	1.77	1.63	(1.06, 2.51)	0.03
OPN	66	66	1.77	1.56	(1.13, 2.15)	0.01
C-peptide (ln)	66	66	1.77	0.62	(0.39, 1.00)	0.05
PD1 (ln)	66	66	1.77	0.82	(0.53, 1.25)	0.35
IL6 (ln)	66	66	1.77	1.26	(0.91, 1.75)	0.17
OPG (ln)	66	66	1.77	0.97	(0.84, 1.11)	0.63

*PSA* prostate specific antigen, *RANK* Receptor Activator of Nuclear Factor k B, *IGFBP* insulin-like growth factor binding protein, *IGF* insulin-like growth factor, *HGF* hepatocyte growth factor, *OPN* osteopontin, *PD1* Programmed cell death protein 1, *IL6* interleukin-6, *OPG* Osteoprotegerin

For continuous measures, the HR is based on a one unit difference in the measurement

The multivariable CART model adjusted by treatment and metastatic volume found a cutpoint for insulin only. However, CART models including the biomarkers only identified cutpoints for HGF and OPN. Cumulative Martingale Residual assessment confirmed that these 3 markers need to be included as dichotomous variables in the multivariate model to avoid bias. Table 3 presents the multivariable model results for PFS. When accounting for metastasis volume, treatment, HGF, OPN, and C-Peptide having Insulin levels below 2.1 (ln scale) more than doubled the risk of progression or death (HR = 2.55 [1.24, 5.23]; *p* = 0.011). Similarly, patients with HGF levels higher than 8.9 (ln scale) had double the risk of progression or death (HR = 2.00 [1.08, 3.70]; *p* = 0.027). OPN and C-peptide were not significant at the 0.05 level, but removing them changes the significance of the others, so all markers remain together in the same model. Figure 2a-d represents a risk stratification model for progression using insulin (ln scale below 2.1), HGF (ln scale above 11.9), and OPN (ln scale above 8.9), and C-peptide (below the median = 3309.607). No patient had all 4 risk factors. Thus, four risk groups with distinct PFS curves (*p* = 0.004) were identified, as outlined in Fig. 2e: 0, 1, 2, or 3 risk factors with corresponding median PFS of 9.8 , 2.0, 1.6, and 0.7 years, respectively.

**Table 3:** Multivariable analysis for PFS adjusted by treatment and metastatic volume

	HR	95 % CI for HR	*p*-value
Mets Volume (High vs. Low)	2.66	(1.34, 5.27)	0.005
Treatment (ADT + Chemo vs. ADT)	1.09	(0.65, 1.84)	0.75
Insulin (ln)^a^ (<2.1 vs. ≥2.1)	2.55	(1.24, 5.23)	0.011
HGF (ln)^a^ (>8.9 vs. ≤8.9)	2.00	(1.08, 3.70)	0.027
OPN (ln)^a^ (>11.9 vs. ≤11.9)	1.67	(0.84, 3.33)	0.147
C-peptide (ln) (Unit = 1000)	0.97	(0.86, 1.10)	0.622

*OPN* osteopontin, *HGF* hepatocyte growth factor

^a^Cut points were determined by CART analysis

## Discussion

The emergence of castration-resistance signals the lethal potential of metastatic prostate cancer and is considered to be a biologically and clinically significant event. Considerable research effort is currently directed towards the development of novel therapy strategies that delay the onset of castration-resistance and/or overcome mechanisms of castration-resistance [1]. Thus, the current inability to predict when an individual patient will develop castration-resistant disease confounds clinical practice, challenges personalization of therapy, and fosters uncertainty for both patients and physicians. Based on this, there is a great need to identify pre-treatment prognostic markers for the development of castration-resistant disease with the goal to anticipate this event and direct vulnerable patients towards novel therapy strategies.

In this study, we initially sought to test a biologically derived hypothesis that baseline insulin levels with or without other markers would predict time to castration-resistant progression in patients with advanced prostate cancer treated with ADT. By multivariable analysis, we found that higher insulin levels were associated with longer time to castration-resistant progression in our cohort. Because higher insulin levels are indicative of metabolic syndrome and both have previously been associated with increased risk of developing and dying from prostate cancer, we initially predicted that higher insulin levels would also correlate with shorter time to castration-resistant progression. In support of this, Flanagan et al. recently found that the presence of metabolic syndrome (defined by multiple criteria of which hyperinsulinemia is one) is a risk factor for the earlier development of castration-resistant progression in patients with metastatic androgen-dependent prostate cancer treated with ADT [23]. However, in their study, insulin levels were not specifically evaluated, and more importantly, they showed that higher glucose levels (i.e. by inference lower relative insulin secretion), was associated with shorter time to castration resistance. This is actually in keeping with our findings. Although our retrospective data set did not permit characterization of metabolic syndrome in individual patients, our findings suggest complex interactions between insulin and energy metabolism versus prostate cancer development, and prostate cancer progression. It is very important to view our seemingly contrary results in view of the published literature, including some recent new studies. Previous work has proposed hyperinsulinemia as a risk factor for lethal clinical prostate cancer [24]. However, it is important to note that this association was only observed in men with newly diagnosed localized prostate cancer, not men with more advanced metastatic setting, as included in our study. A more recent meta-analysis of more than 10,000 men with prostate cancer established a positive correlation of IGF-1 and 2 with prostate cancer development, but not progression of metastatic disease [25]. Perhaps the differential role of insulin and IGF-1 in prostate cancer development vs. castration resistance is one of the underlying reasons for the failure of cixutumumab (a monoclonal antibody that targets IGF-1R) to improve time to castration resistance in metastatic prostate cancer [26] (an almost identical cohort to our patient cohort).

Furthermore, a large study of the European Prospective Investigation into Cancer and Nutrition (EPIC) showed that men with diabetes had a 26 % lower risk of prostate cancer [27]. Consequently, only metformin has been associated with reduced risk for prostate cancer, while oral hypoglycemia that directly affect insulin, have not [28]. The postulated anti-tumor effects of metformin are complex, but they appear not to be insulin mediated, but possibly through inhibition of cancer stem cells [29].

Obviously, the fact that blood samples from our study were not uniformly collected with respect to fasting versus non-fasting may have confounded our results. It would have been potentially beneficial to correlate insulin levels with other parameters of insulin resistance to further corroborate our findings. However, such markers were not part of the tested biomarker panel and lack of sample availability for further testing means this option should be pursued in an independent cohort validation. Despite the fact that we don’t have certainty on the fasting status of the patients at the time of blood draw, we note that the half-life of endogenous insulin secreted by pancreatic beta cells in response to glucose stimulation is about 4 min [30], i.e. >99 % of the endogenous insulin is expected to be degraded after 30 min (i.e. seven half lives). Thus, on a practical level (e.g. the patient flow at the cancer center which includes check in, waiting at the lab, followed by blood draw, which usually takes longer than 30 min), it is likely that the measured insulin levels reflect the subject’s individual baseline levels, an assumption further supported by the corresponding C-Peptide levels in our study.

Biologically, one plausible explanation for our data is that hyperinsulinemia cooperates with ADT to suppress autocrine-paracrine sources of testosterone and this dual inhibition may delay progression to castration-resistance. It has previously been shown that pre-existing hyperinsulinemia (for example with type II diabetes, central adiposity, and/or dyslipidemia) is associated with reduced systemic testosterone levels, likely through suppression of adrenal androgens [15]. We, and others, have previously shown that the transition from androgen dependence to castration-resistance is accompanied by a shift in testosterone synthesis from gonadal (endocrine) to non-gonadal sources (autocrine-paracrine) [31, 32]. Within the bone-tumor microenvironment, adrenal androgenic precursors are converted to testosterone. Since ADT only blocks endocrine sources of testosterone, hyperinsulinemia might suppress autocrine-paracrine sources of testosterone to enhance therapy benefit. Additional studies will be required to test this possibility.

Thus, in summary, while our original hypothesis on the role of insulin was based on an extrapolation of published data in risk of developing and aggressiveness of non-metatstatic prostate cancer, our actual findings are more in keeping with recent literature regarding members of the insulin family hormones and castration-resistance.

We also sought to develop a discovery platform to identify other potentially important serum factors implicated in castration-resistant progression. Towards this goal, we identified elevated (i.e. above the CART cut-off) levels of OPN and HGF and lower levels of C-Peptide as individually associated with reduced PFS. HGF, and its cell surface receptor (aka MET kinase), are well described in progression and invasion of several tumor types, including prostate cancer [33]. Met inhibitors such as the tyrosine kinase inhibitor cabozantinib are already in clinical trials for advanced prostate cancer [1] and recent trials have shown high MET expression is associated with inferior survival [34]. OPN, a ligand for CD44, intergrins, and other cell surface receptors, is implicated in tumor progression [35], and has been shown recently to correlate with response to chemotherapy in metastatic CRPC [36], and in a meta-analysis was significantly associated with survival in several cancers, including prostate cancer [37]. When we combined all four risk factors, we obtained a better separation of PFS based on the patients’ numbers of risk factors. Thus, our model of risk stratification is based on serum proteins that mechanistically contribute to the complex biology of castration-resistant progression.

The available samples for analysis represented only a subset of the originally recruited patients since this was a retrospective study and prospective sample collection was not part of the original protocol. The large number of variables, the cutpoint determination and variable selection may have resulted in over-fitting. However, the biomarkers included in the final model all have reported biologic rationale in the literature, which somewhat increases the confidence in their validity for this specific question This work indicates that further study of these markers is warranted.

Taken together, we are reporting that a novel prediction model for patients with metastatic androgen-dependent prostate cancer who are receiving ADT may be within reach. The exploratory nature of our study requires additional independent validation of biomarkers with robust methods in a larger group of patients to establish a more reliable risk grouping. Even so, if confirmed, this model may potentially enhance our limited prognostic tools available for this patient population.

## Conclusions

Pretreatment serum insulin, HGF, OPN, and C-peptide levels can predict PFS in men with mADPC treated with ADT. Independent validation of this algorithm could provide a novel prognostic tool for patients with mADPC.

## Abbreviations

ADT: Androgen deprivation therapy
CI: Confidence interval
CRPC: Castration-resistant prostate cancer
HGF: Hepatocyte growth factor
HVM: High-volume metastasis
HR: Hazard ratio
IGF-1R: Insulin-like growth factor-1
IR: Insulin receptor
LVM: Low volume metastasis
OS: Overall survival
PFS: Progression-free survival
mADPC: Metastatic androgen-dependent prostate cancer
OPN: Osteopontin
PSA: Prostate specific antigen
RF: Risk factors
